# P-445. Clinical Complications and Quality of Life in People Living with HIV (PLWH) and Other Comorbidities

**DOI:** 10.1093/ofid/ofae631.645

**Published:** 2025-01-29

**Authors:** Grace Lui, Catherine Cheung, Ka-Ki Yuen, Vivian Wong, Chuk-To Au-Yeung, Hang-Yee Ho, Timothy Li

**Affiliations:** The Chinese University of Hong Kong, Hong Kong, Hong Kong; The Chinese University of Hong Kong, Hong Kong, Hong Kong; The Chinese University of Hong Kong, Hong Kong, Hong Kong; The Chinese University of Hong Kong, Hong Kong, Hong Kong; The Chinese University of Hong Kong, Hong Kong, Hong Kong; The Chinese University of Hong Kong, Hong Kong, Hong Kong; The Chinese University of Hong Kong, Hong Kong, Hong Kong

## Abstract

**Background:**

Comorbidities in PLWH tend to cluster, and may raise risk of multiple clinical complications. It is important to understand the burden of these complications and health-related quality of life (QOL) in PLWH with comorbidities.

**Figure:**
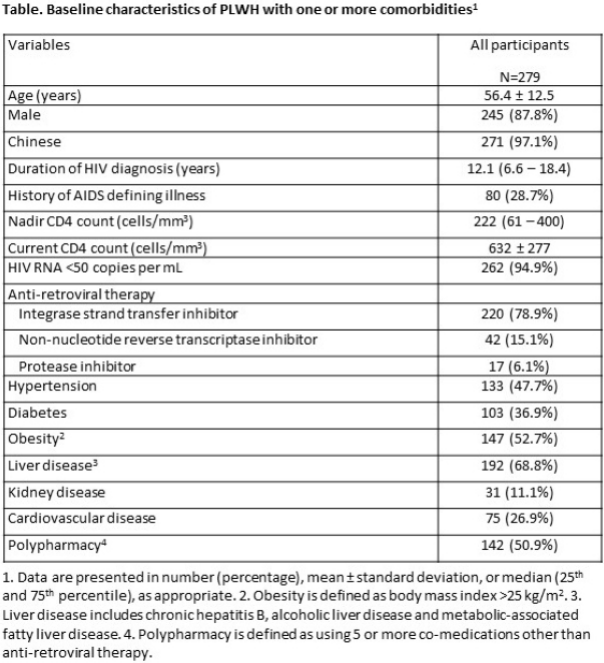

**Methods:**

This is an ongoing prospective longitudinal observational study in Hong Kong to determine the prevalence and incidence of clinical complications among PLWH with ≥1 documented comorbidities (hypertension, diabetes, obesity, liver disease, kidney disease and cardiovascular disease). The endpoints are the prevalence of each clinical complication and QOL. Clinical complications are (i) high cardiovascular risk (QRISK3 >10%), (ii) renal complication (glomerular filtrate rate < 60 mL/min/1.73m^2^, or urine albumin:creatinine >3 mg/mmol), (iii) liver steatosis and significant liver fibrosis (Controlled Attenuated Parameter >248 dB/m and liver stiffness >9.0 kPa, measured by transient elastography) and (iv) low bone mineral density (BMD) at lumbar spine and femoral neck (T score < -1.0 if ≥50 years or Z score < -2.0 otherwise, measured by dual energy X-ray absorptiometry). QOL was measured by EQ-5D-5L. Association between each comorbidity and the endpoints are determined by logistic or linear regression analyses, adjusted for age and gender.

**Figure:**
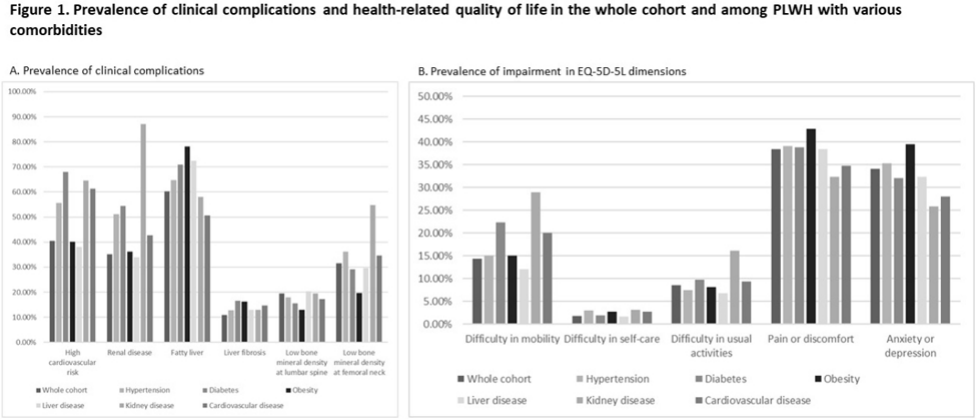

**Results:**

We included data from the first 279 PLWH (baseline characteristics in Table). Overall, 40.5% had high cardiovascular risk, 35.1% had renal complication, 10.8% had significant liver fibrosis, and 31.5% had low BMD at femoral neck (Figure 1A), while 14.3% had difficulty in mobility, 38.4% had pain or discomfort, and 34.1% had anxiety or depression (Figure 1B). EQ VAS was 80 (25^th^, 75^th^ percentile 67, 85).

PLWH with hypertension and diabetes had significantly higher cardiovascular risk and renal complication. Diabetes and obesity were significantly associated with liver fibrosis, but lower risk of low BMD (Figure 2).

PLWH with diabetes had significantly greater impairment in mobility, while obesity was associated with a trend of pain or discomfort and anxiety or depression (Figure 3). Kidney disease was associated with worse EQ VAS (unstandardized B -7.032, p=0.022).

**Figure:**
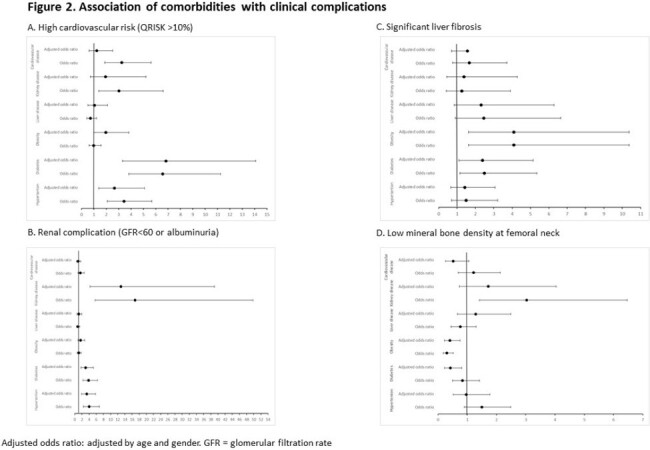

**Conclusion:**

PLWH with comorbidities are vulnerable for multiple clinical complications and worse QOL.

**Figure:**
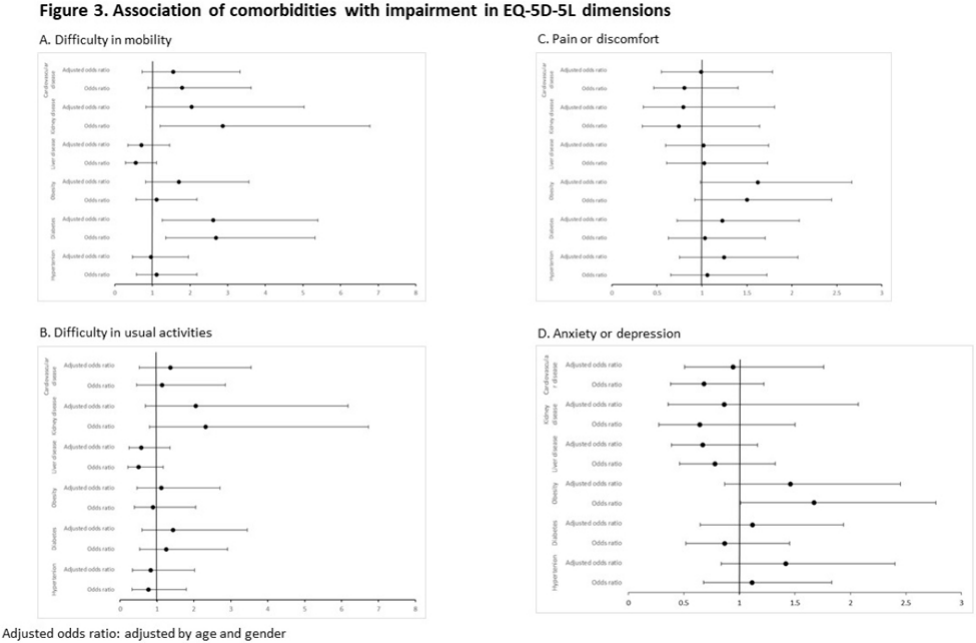

**Disclosures:**

**Grace Lui, MBChB**, Gilead Sciences: Grant/Research Support|MSD: Grant/Research Support

